# Product News

**DOI:** 10.1155/S1463924680000168

**Published:** 1980


PSC-7 Guidelines for Kinetic Analysis of Enzyme Reactions.

PSEP-2 Protocol for Establishing Performance Claims for Clinical Methods – Introduction and Performance – Check experiment.

PSEP-3 ibid – Replication Experiment.

PSEP-4 ibid – Comparison of Methods Experiment.

PSC-12 Definitions of Quantities and Conventions related to Blood pH and Gas Analysis.

TSC-5 Methodological Principles for establishing Principal assigned values to Calibrators.

### Swansea summer school of automatic chemical analysis

July 6–11 1980 University College Swansea.

An intensive short course covering all aspects of automatic analysis will be held in Swansea. The programme will comprise of a series of authoritative lectures given by a team of world experts in automatic chemical analysis, tutorial sessions and practical hands on experience of some of the latest automatic instruments. In addition students will consider the automation strategy necessary to solve a real world course problem. The following experts in automation have already indicated their attendance, Professor H.L. Pardue, Professor M. Bonner Denton, Professor J. Ruzicka, Dr. J. Betteridge, Dr. F.L. Mitchell, Dr. P. Saunders, Dr. D. Deans, Dr. P.B. Stockwell, D.G. Porter and J.K. Foreman. For further details of this course suitable for both practising chemists and managers alike please write to Dr. J. Betteridge, University College of Swansea. Course fee is £270 – early booking necessary due to limited accommodation.

### Programming microprocessors for industrial measurement and control

A two-day seminar on Programming microprocessors for industrial measurement and control is being jointly organised by Sira and Warren Spring Laboratory. It will be held on 29-30 April 1980 in London.

The aims of the seminar are to clarify the various classes of need for programming, in relation to the several levels of programmability that should be provided by the new generation of microprocessor-based products, to present the views of the protagonists of assembly level and of high level languages, and to draw some conclusions regarding the best approach to adopt in programming microprocessors at each of the three stages: product development, installation and in-use operation. The programme comprises eight sessions including software and programming techniques, device programming, system programming and validity and testing of software.

*Enquiries should be sent to Mrs R G Keiller, Sira Institute Ltd, South Hill, Chislehurst, Kent BR7 5EH, UK.*

### Product News

### Capillary dispenser

The Camag capillary dispenser system offers the high precision of sample positioning that is required for automatic chromatogram scanning.

It is loaded with a disposable capillary by pushing the pipette holder into the mouth of the dispenser. Once filled with sample solution the holder is placed in the magnet head of their nanomat from where it is dropped on to the layer surface at an adjusted speed and for a regulated contact time. With the automatic repetition device of the nanomat the capillary content can be delivered in small increments. Pipette holders and capillaries in dispensing magazines are available in sizes ranging from 0.5 - 5$\mu$l.

*Camag, Homburgerstrasse 24, CH-4132 Muttenz, Switzerland.*

### Cassette data recorder

A compact, portable and weatherproof recorder, the CR50, which records on standard cassettes has been introduced by Grant Instruments. The recorder has been used to monitor conditions in the transportation of refrigerated food and as a remote unattended weather recorder. Inputs can be in the form of resistance, voltage, pulse counting or digital information. Sensors which can be used with the recorder include thermistors, platinum resistances, thermocouples, wet and dry psychrometers and relative humidity sensors. The recorder is designed to work in a range of temperature from -20 to +60°C. Standard type cassettes are used, together with easily available standard batteries.

On every cycle a recording is made from each sensor in turn, followed by a recording of real-time and an identification number. Cycles are initiated at intervals which can be pre-set by the user between 1 and 999 minutes on a 3-digit thumbwheel switch. Completion of a recording cycle takes under 2 seconds.

*Grant Instruments (Cambridge) Ltd, Barrington, Cambridge CB2 5QZ, UK.*

### Radiation contamination monitor

Laboratory Impex have recently introduced a new high sensitivity beta and gamma contamination monitor. The instrument, the LB1210B, offers a detection limit of 10 $^{-5}$ uCi/cm$^2$. The 100 cm$^2$ xenon filled detector fitted to the monitor is designed to survey either rough or smooth surfaces and can be adapted for use as an exit or atmosphere monitor without loss of sensitivity due to temperature or humidity fluctuations. The detector can be operated single-handed. Radiation levels are indicated on a large meter scale with x 1 or x 10 time constant readout modes. The x 10 mode operates a 20 second integrate cycle giving high measurement precision. A built in audible alarm with an adjustable threshold indicated the presence of a contaminated area and low battery power is indicated by an LED warning light. The unit can be operated direct from a 220/240 volt mains supply.

*Laboratory Impex Ltd, Lion Road, Twickenham, Middx, UK.*

### Trace element analysis

The plasma emission spectrometer Spectraspan III from Techmation performs simultaneous multi-element analysis to determine trace elements in stainless steels. Sample preparation for most analyses consists of dissolution in the minimum amount of 1:1 HCl/ $HNO_3$ necessary and dilution to a concentration of 2% is sufficient. An echelle grating spectrometer with a resolution claimed to be 10x better than other atomic analysis instruments is incorporated and it permits the use of previously avoided spectral lines such as the niobium 309.4 nm line which has closely associated water bands. The instrument has many applications in the analysis of wear metals in heavy engineering and aircraft industries.

*Techmation, Ltd, 58 Edgware Way, Edgware, Middx, UK.*

### UV/vis spectrophotometer

The 8450A uv/vis spectrophotometer is a recent addition to Hewlett Packard's range of analytical instrumentation. The spectrophotometer has a 16-bit 44 K-word microcomputer and can analyse up to 12 components simultaneously, seven over the full wavelength range. A CRT display allows the complete spectrum to be viewed. The HP-1B and RS232 interfaces permit the spectrophotometer to be coupled to other instruments and special interface hardware and software are avoided. Peripherals include a graphics plotter, a plotter/printer, and a cartridge tape unit. Spectral data processing, measurement of light-sensitive materials, mixture analyses aids process control, enzyme kinetic measurements and quality control of pharmaceuticals are a few of the applications of the spectrophotometer.

*Hewlett Packard Ltd, King Street Lane, Winnersh, Wokingham, Berkshire, UK.*

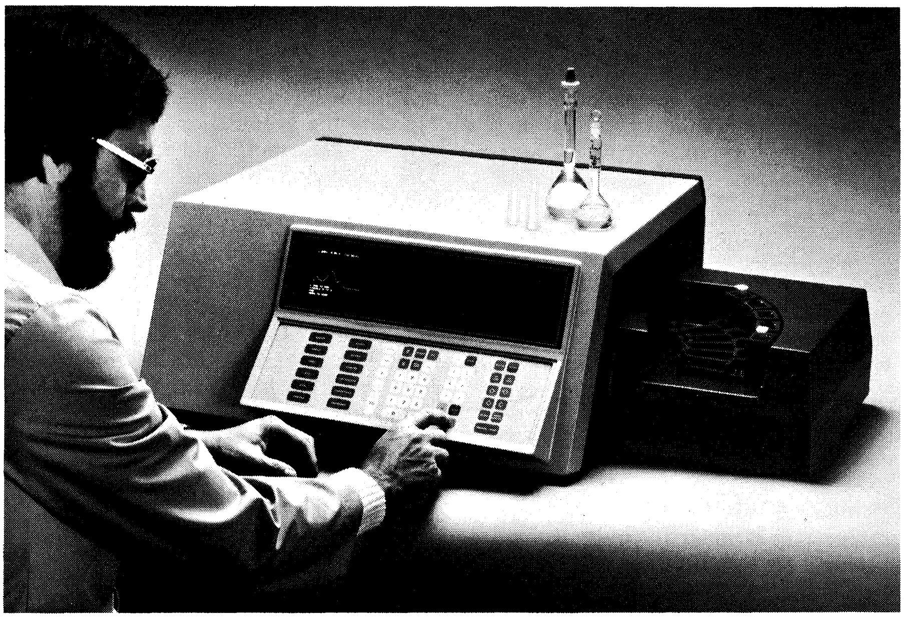

*Hewlett Packard's 8450A spectrophotometer*

### Gas chromatograph system

A computer-based process gas chromatograph system for high accuracy sampling and analysis of gases involved in refining and the production of petrochemicals and chemicals, has been introduced by the Environmental and Process Instruments Division of Bendix Corporation. The equipment comprises an analyser and a chromatograph controller which combine to give a facility which can be tailored to meet customers' individual requirements. The analyser can be used in hazardous areas. Features of the equipment include a low-mass, stainless steel-lined oven and heater designed for quick temperature recovery and control, a lockable air purge system for the electronics enclosure, external manual switching and optional serial output. A proportional temperature controller provides accurate selection of air bath oven temperature over the range 50 °C to 220 °C. The controller handles all output value commands and the input analog signals. The control panel may be located remotely.

Optional features include trend output for eight separate voltage signals, digital outputs to control stream valve or other contact closure requirements (maximum of 20), and serial transmission in ASCII code to a teleprinter, CRT or host computer.

*Horiba Instruments Ltd, 5 Harrowden Road, Brackmills, Northampton NN4 OEB, UK.*

### Ultrasonic disintegrators

Ultrasonics produces a wide range of ultrasonic disintegrators, suitable for many applications where various levels of intense disruptive energy are required to disintegrate cells and microorganisms and to accelerate enzymatic activity and chemical reactions. The normal probe type models are complemented by the continuous flow models which enable a greater quantity of material to be uniformly treated. A continuous flow model comprises a generator and a cabinet which houses a disintegrator head and a variable speed pump. The treatment chamber is made from stainless steel and a heating/cooling jacket is fitted to ensure a constant temperature. The material is fed via the pump through the treatment chamber at a regular distance from the surface of the probe face, thus reducing the possibility of localised currents.

*Ultrasonics Ltd, Otley Road, Shipley, West Yorkshire, UK.*

*Continuous flow ultrasonic disintegrator*

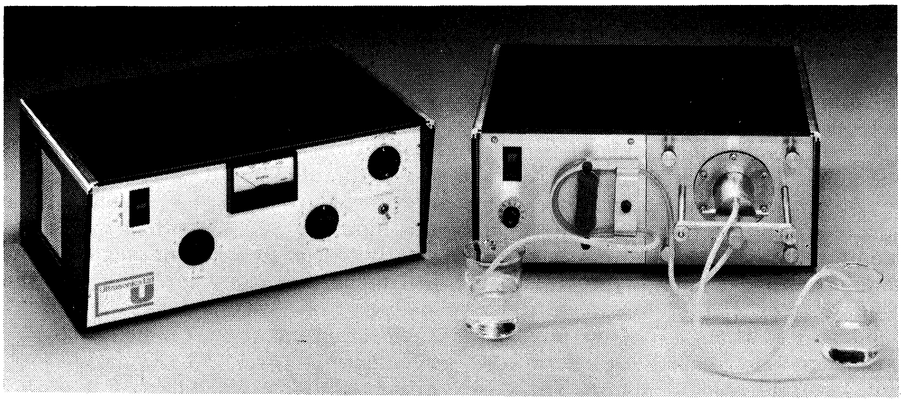

### Universal concept terminal

Vector International have introduced the universal concept terminal, a modular system which enables the OEM to offer terminal-oriented products that can be operated by unskilled personnel. Current applications include laboratory data collectors, processors, industrial control devices and a tank monitoring system. The basic system features a 74-key keyboard, two 9-digit displays, a 40-column alphanumeric matrix printer, a six-position keylock, micro-computer, power supply and five Eurocard slots for function expansion. The keyboard module includes an interface card for the microprocessor bus and a software routine to read the ASCII codes generated by the keys. The display module features an interface card and control software.

Optional features include a mini-floppy drive, system battery backup for 1K memory, battery-protected clock and calendar, programmable parallel I/O, opto-isolated parallel input and analog-digital conversion. The minifloppy module provides 80.6 K bytes of random access bulk data. The software also uses a modular approach and features an IOCS program package which includes interface subroutines for all devices in the system. The application-level program can be written in assembly, PL/M, Fortran or Basic.

*Comark Europe, Chaussee de Charleroi 27, B-1060 Brussels, Belgium.*

### Automated control for HPLC

The chromatograph control module, introduced by LDC, is controlled by a single microprocessor-based unit with a built-in data handling system. Multiple programmed runs may be stored in the unit's memory allowing long-term unattended operation. The module connects to any one of LDC's liquid chromatography range. A full alpha-numeric keyboard allows access to the computer and visual output on a shielded display screen shows instructions, analysis status and parameter files in plain language rather than in mnemonic codes. A digital tape cassette system and the provision of LDC BASIC gives the CCM minicomputer capability.
*Laboratory Data Control, Shannon International Airport, Ireland*

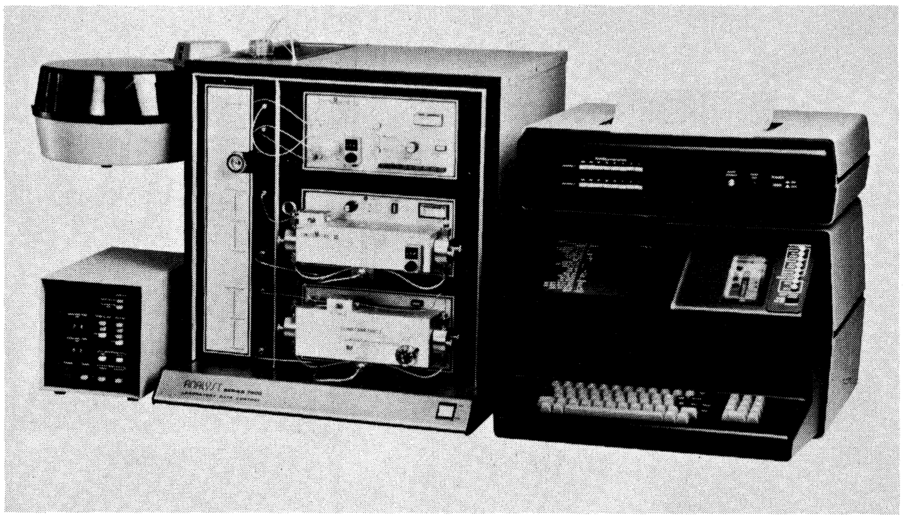

*LDC chromatograph control module*

### Optoacoustic spectrometer

EDT Research have announced the availability, as an option of the OAS 400 optoacoustic spectrometer of an RS232 serial output interface. Data on absorption intensity and wavelength will be available digitally through this interface for feeding into a mini-, micro- or a mainframe computer for storage and subsequent processing. A dedicated computer-based data terminal is under development.
*EDT Research, 14 Trading Estate Road, London, UK.*

### Autosampler

A microprocessor-controlled sampling system from C.I.Electronics has been designed for dissolution testing. The unit contains ten switch-selectable programs to sample from six vessels three to five times at precise intervals in total periods of 30 minutes - 8 hours. The system comprises a 20-channel peristaltic pump, a mechanical unit for sample handling and collection and an electronic controller. The mechanical unit is encased in stainless steel and carries the controller above the level of liquid handling. The controller front panel has a thumbwheel switch for program selection. It provides an option for leaving some sample cups empty in order to permit subsequent addition of standard solutions to a sample series. The controller contains an elapsed time indicator and audible warning of program completion.
*C.I.Electronics Ltd, Brunel Road, Churchfields, Salisbury, Wilts, UK.*

*Microprocessor-controlled sampling system for dissolution testing*

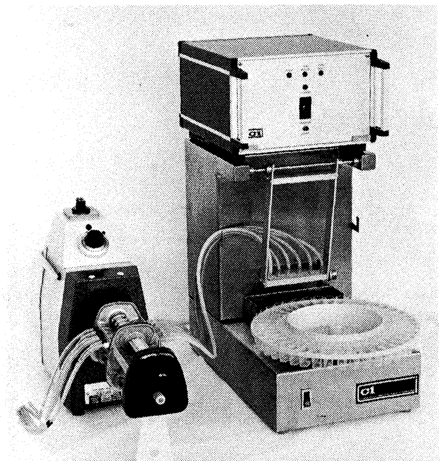

### Dielectric measurements

ChemLab Instruments have added dekameters and dipolmeters to the range of WTW equipment they market in the UK. The dekameter provides rapid measurement of dielectric constant and dielectric loss factor of liquids, emulsions, oils, powders. It can be used for single sample measurement in the laboratory, or for continuous measuring as part of a process control unit. The diplometer is a compact instrument, designed for the measurement of the electrical dipole moment in. molecular structures. It can also be used to measure the dielectric constant of materials and for the quantitative analysis of substances such as volatile oils. A range of measuring cells is available for use with both these instruments.
*ChemLab Instruments Ltd, Hornminster House, 129 Upminster Road, Hornchurch, Essex, UK.*

### Thermo-titrator

Sanda has developed a thermo-titrator which is a semi-automatic thermometric titration device. It utilizes thermal effects, as commonly encountered in acid-base titrations, for end-point detection. The thermo-titrator can operate both aqueous and non-aqueous since its method of location of equivalence point in titrimetry is based on the heat of reaction. Where there is a pK difference of 2, separations are made between similar compounds while with other methods a pK value of 4 is required. It has one universal probe which does not have corrosion reaction problems. A classical calorimetric curve, a derivative curve or a derivative with end-point are modes of operation.
*Sanda Inc, 4343 East River Drive, Philadelphia, Pennsylvania 19129, USA.*

### Batch analyser

The Clinicon Corona is a compact fully automated batch analyser which will determine enzymes, substrates and proteins by constant rate, fixed time or end point methods. All analytical procedures and parameters are under the control of a microprocessor system to ensure simple operation. It embraces a profile of predetermined analysis parameters for the major areas of interest to the clinical chemist. It is also possible to adapt existing methods, develop new methods or adopt future methods.
*Clinicon Ltd, Bell Lane, Lewes, Sussex, UK.*

### Multichannel analysers

The Astra 4 and Astra 8 automated stat/routine analyser systems from Beckman incorporate computer control and monitoring for accurate testing of electrolytes and routine chemistries. There are six chemistry modules currently available.

The built-in microprocessor allows advanced programming of up to 80 samples and programming is also possible during a chemistry run without loss of results. Computer/operator communication on the Astra 4 is achieved by a printer and on the Astra 8 by means of a video screen or printer. Other features include 24 hour availability, ease of operation, accuracy, and small sample volume for seven determinations.
*Beckman RIIC Ltd, Turnpike Road, Cressex Industrial Estate, High Wycombe, Bucks, UK.*

### Organics-in-water analysis

The Finnigan organics-in-water gas chromatograph/mass spectrometer system has been designed to meet the needs of industry in monitoring and analysing industrial waste water. The system is easy to operate and automatic tuning with a specific program optimizes the mass spectrometer to meet EPA tuning requirements. Automated software routines are provided to make analyses rapid and precise. Data acquisition can occur simultaneously with data processing to increase sample throughput. Standard output capabilities include extracted ion current profiles, total ion profiles, library searches, single spectrum displays and complete quantitation reports. The system includes a fully automated Perkin-Elmer Sigma series microprocessor controlled gas chromatograph.

*Full details are provided on a data sheet available from Finnigan Corporation, 845 West Maude Avenue, PO Box 459, Sunnyvale, California 94086, USA.*

### Hydride generation system

The development of a custom-designed hydride generation system from Techmation has enabled the SMI Spectraspan range of plasma emission spectrometers to be used for the determination of ultra trace levels of As Sb, Se, and other hydride producing elements difficult to detect by conventional techniques. The system provides a 10-100 fold increase in sensitivity over direct nebulisation for As and Se. It can also be used for Hg vapour production for low level Hg determination. The hydride generation assembly consists of a reaction cell, drying tube, hydrogen delay column, flow meter and valves. The standard Spectrajet III ceramic sample tube is replaced by a special sample introduction tube.

*Techmation Ltd., 58 Edgware Way, Middx, UK.*

*Multitask spectrometer from Kevex*

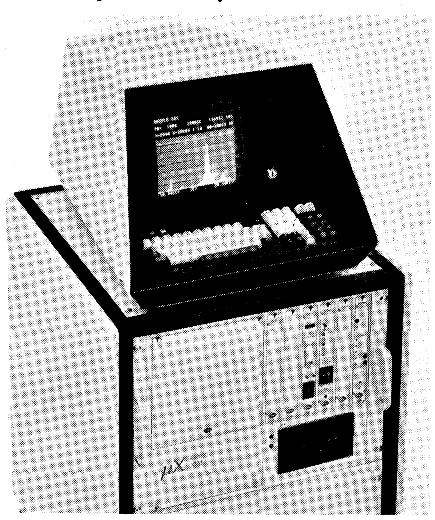

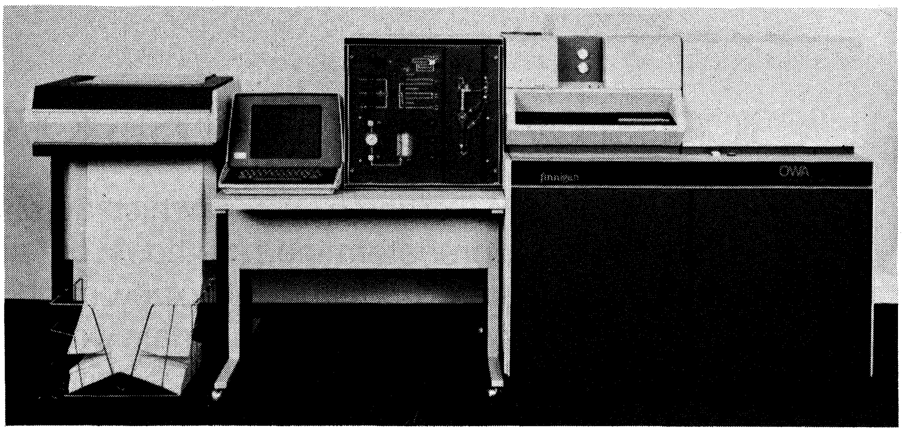

*Finnigan organics-in-water mass spectrometer system*

### Spectrometers

Unispec analyzers are a series of multitask spectrometers from Kevex Corporation with data handling capabilities for x-ray energy, Auger, photoelectron, secondary ion mass and ion sputtering spectroscopies. The basic system includes a mainframe housing data storage memory, standard firmware and signal processing electronics. An integrated control console combines a 63 key ASCII keyboard with colour-coded video display. Data quantitation capability for x-ray analysis may be augmented with 14 supplementary firmware routines including thin film and multiple regression analysis.

*Analytical Instrument Division, Kevex Corporation, 1101 Chess Drive, PO Box 4050, Foster City, California, 94404, USA.*

### Thermal analyser

A simultaneous thermobalance-differential thermal analyser for the measuring of weight and thermal changes of materials has been introduced by Stanton Redcroft. The equipment incorporates microprocessor control and allows the physical and chemical phenomena of many different materials to be studied. Other products from Stanton Redcroft include temperature programmers, thermobalances, a hot stage microscope attachment, a thermo mechanical analyser and mineral insulated thermocouples.

*Stanton Redcroft, Copper Mill Lane, London SW17 0BN, UK.*

### Microprocessor applications trainer

Recently launched by Feedback Instruments, the microprocessor applications trainer MAT385 has been specifically designed for easy comprehension of the potential of the microprocessor as a versatile tool in many situations. The trainer has been conceived to provide a full course of study including an introduction to microprocessor concepts together with experience of numerous real applications, with practice in adapting and writing programs to fulfil different requirements and fresh applications.

The trainer is self-contained with all the essential facilities and software needed to put the system into service and a comprehensive instructional handbook and a machine code reference booklet.

*Feedback Instruments Ltd, Park Road, Crowborough, Sussex TN6 2QR, UK.*

### Digital panel meters

John Minister Instruments have announced the availability from Velonex, Santa Clara, of a new range of miniature panel meters, model 371 with large characters. The meters have a low power consumption, of less than 40 milliwatts, which allows the meter to be used in portable applications. No zero adjustment is necessary and the meters have a 2 year guarantee.

The overall dimensions are 3.01 x 1.52 x 0.75 inches.

*John Minister Instruments Ltd, 137/139 Sandgate Road, Folkestone, Kent CT20 2DE, UK.*

### Density determination

An automatic helium pycnometer, requiring no operator attention after sample loading is available from Micromeritics Instrument Corporation. The pycnometer provides density determinations in less than 20 minutes. Using microprocessor technology, sample mass is entered directly into the instrument memory, the dry sample is loaded and the tune switch is actuated. At the end of the cycle, sample density is displayed on a digital read-out. Applications for the instrument include organic and inorganic pigments, aluminas, silicas, and chromium carbide.

*Coulter Electronics Ltd (UK Agent), Coldharbour Lane, Harpenden, Herts, UK.*

